# Oncocytic adrenocortical neoplasm of borderline uncertain malignant potential diagnosed after robot-assisted adrenalectomy case report

**DOI:** 10.1186/s12894-023-01238-1

**Published:** 2023-04-15

**Authors:** Chih Peng Chin, Ralph Grauer, Burak Ucpinar, Mani Menon, Qiusheng Si, Ketan K. Badani

**Affiliations:** 1 Department of Urology, Icahn School of Medicine at Mount Sinai, 1 Gustave L. Levy Place, New York, NY 10029 USA; 2 Department of Pathology, Icahn School of Medicine at Mount Sinai, New York, NY USA

**Keywords:** Adrenal oncocytic tumor, Oncocytoma, Genitourinary neoplasm, Robotic surgery, Oncology

## Abstract

**Background:**

Adrenal incidentalomas are radiologically discovered tumors that represent a variety of pathologies, with the diagnosis clinched only on surgical pathology. These tumors may be clinically monitored, but triggers for surgery include size > 4 cm, concerning features on radiology, or hormonally functioning. Adrenal oncocytic neoplasms (AONs) are notably rare and typically nonfunctional tumors that are discovered as incidentalomas and exist on a spectrum of malignant potential.

**Case presentation:**

We discovered an exceptionally large (15 cm in the greatest dimension) incidentaloma in a 73-year-old man with left back pain and he was treated with robotic-assisted adrenalectomy. Surgical pathology was consistent with AON of borderline uncertain malignant potential; adjuvant mitotane and radiation were omitted based on shared decision-making.

**Conclusion:**

Large AONs are rare, usually benign tumors that can be safely treated with robotic-assisted adrenalectomy. Surgical pathology is the crux of diagnosis and post-operative management, as it informs both the initiation of adjuvant therapy and the stringency of post-operative surveillance.

**Supplementary Information:**

The online version contains supplementary material available at 10.1186/s12894-023-01238-1.

## Highlights

- Oncocytic adrenocortical neoplasms (OANs) are rare tumors of the adrenal gland that span the clinical continuum from benign to malignant.
- Large (> 4–6 cm) OANs are indistinguishable from adrenal cortical carcinomas preoperatively and are only diagnosed via surgical pathology following open, laparoscopic, or robotic adrenalectomy.
- Given aggressive features on pathology, adjuvant treatment with mitotane, chemotherapy, and/or radiation is considered.
- In the absence of high-risk pathology, surveillance is an option with CT scans every 3 months for up to 5 years.

**Supplementary Information:**

The online version contains supplementary material available at 10.1186/s12894-023-01238-1.

## Background

Adrenal incidentalomas larger than 4 cm are at risk of being poorly differentiated adrenal cortical carcinomas (ACCs) and treatment is recommended with either open, laparoscopic, or robotic adrenalectomy [1]. Infrequently, these large tumors are diagnosed as benign entities on surgical pathology. Adrenal oncocytic neoplasms (AONs) are eosinophil-rich tumors radiologically indistinguishable from ACC; thus, diagnosis depends on tissue pathology [2]. AONs are predominantly benign, hormonally nonfunctional, and preferentially affect women (2.5:1) and the left adrenal gland (3.5:1) [2]. AONs are sparsely reported in the literature with approximately 150–200 being described primarily in case reports and limited case series [2]. Herein we describe the management of an AON that is, to our knowledge, the largest ever reported.

## Case presentation

**Fig. 1:**
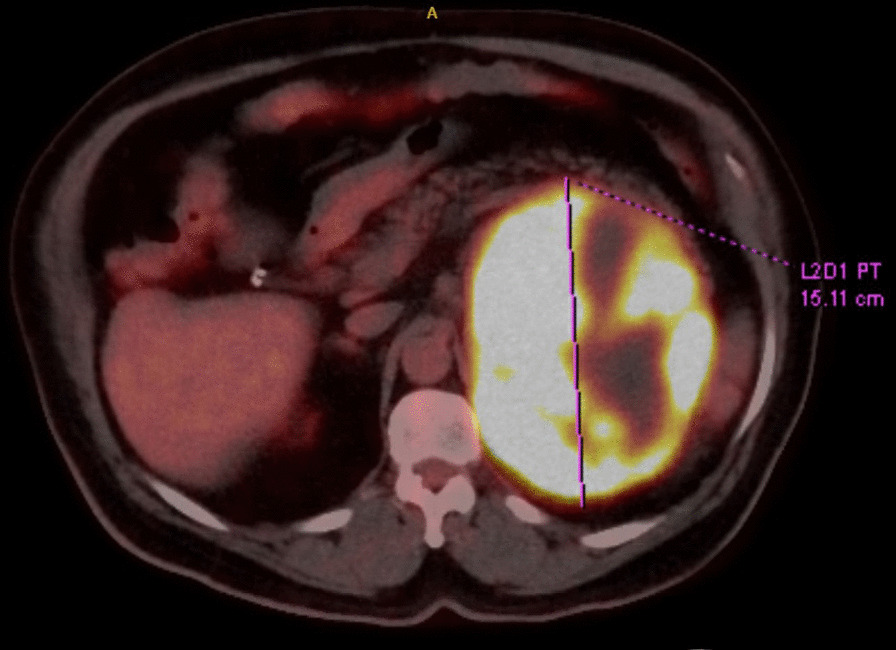
Axial PET scan consistent showing metabolically avid adrenal mass, measuring 15 cm

A 73-year-old man was diagnosed with a left adrenal mass that measured 14.8 × 11.2 × 9.8 cm following a contrast-enhanced abdomen/pelvis CT scan obtained to evaluate left back pain. He reported no flushing, sweating, palpitations, headache, weight changes, or proximal muscle weakness. Subsequent basic metabolic panel, renin and aldosterone, random plasma cortisol and low-dose dexamethasone (1 mg) suppression test, 17-hydroxyprogesterone, testosterone, deoxycorticosterone, androstenedione, adrenocorticotropic hormone, and plasma metanephrines were within normal limits; dehydroepiandrosterone sulfate level was mildly elevated to 474 µg/dL (age-adjusted normal: 31–296 µg/dL). 18 F-fluorodeoxyglucose positron emission tomography (FDG-PET) scan (Fig. 1) revealed a large, markedly hypermetabolic necrotic left suprarenal mass of adrenal origin without other findings of metastatic disease.

**Fig. 2:**
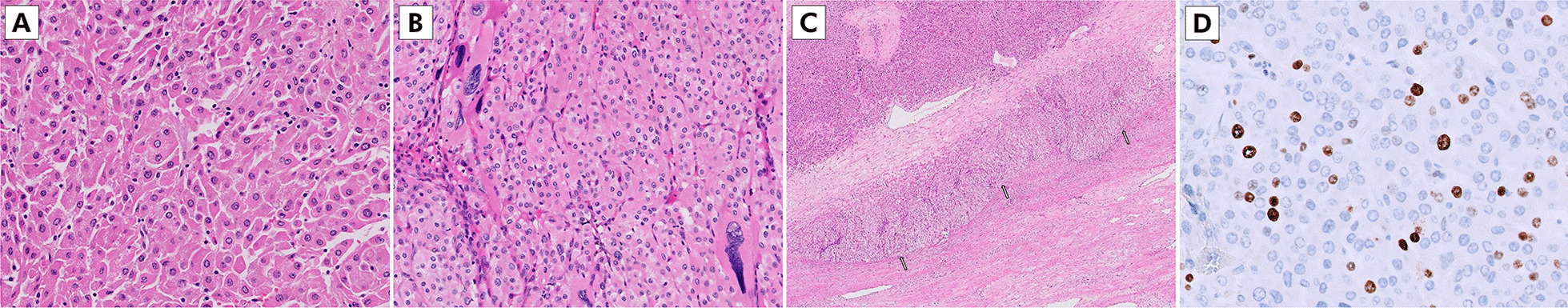
Oncocytic tumor with diffuse growth pattern (**A**, 100×), highly atypical nuclei (**B**, 400×) and capsular invasion (**C**, 20×). The proliferation rate by Ki67 stain is approximately 10% (**D**, 200×)

He underwent a left robotic-assisted transperitoneal adrenalectomy without disruption of tumor capsule (Additional file 1). The excised adrenal containing the encapsulated tumor measured 15.0 × 14.0 × 9.8 cm and weighed 1091 g. The tumor was located 0.2 cm beneath the intact capsule and replaced the entire adrenal gland with no viable adrenal parenchyma grossly identified. The tumor was sectioned once per every centimeter of tumor to ensure appropriate tissue representation. Surgical pathology (Fig. 2) revealed an oncocytic adrenal cortical tumor with diffuse architecture, necrosis, focal nuclear pleomorphism (high nuclear grade), capsular invasion, and proliferation rate by Ki67 of 10–15%. The tumor was negative for p53 immunostaining and beta-catenin was negative in terms of cytoplasmic staining. Surgical margins were negative for tumor and there was no evidence of vascular invasion and no increased mitotic index. The patient was discharged without complications on postoperative day 1. Adjuvant radiation therapy and adjuvant mitotane were considered but shared decision-making resulted in surveillance with CT scans and blood work every 3 months for at least 2 years. At 3 months postoperatively, CT scan was negative for recurrent disease.

## Discussion and conclusions

AONs are diagnosed via surgical pathology following adrenalectomy for presumed malignancy on cross-sectional imaging. The European Society of Endocrinology recommends laparoscopic adrenalectomy for suspected malignant tumors < 6 cm presenting without local invasion [3]. Retrospective evidence supports tumor size as a relative contraindication for minimally invasive adrenalectomy, with the primary concern being incomplete resection or capsular rupture causing local recurrence in large tumors suspected to be malignant [4–6]. However, the NCCN recommends that the surgical approach can be tailored to the surgeon’s technical experience [7]. Robotic surgery allows for better manipulation of fragile adrenal tissue and may decrease the risk of capsular rupture and incomplete resection by virtue of the high-definition three-dimensional view of the operative field and greater range of motion afforded than with laparoscopy or open approach [8]. We opted to perform this case robotically based on the extensive robotic experience of the surgeon and the belief that the potential benefits of decreased postoperative pain and shorter hospital stay vindicated the risks.

The crux of postoperative management in AON is the decision to initiate adjuvant therapy based on the histological criteria assessing malignant potential via either the Lin-Weiss-Bisceglia (LWB) system, reticulin algorithm, or the Helsinki scoring system [9, 10]. The LWB criteria designates an AON as malignant if the tumor meets any of the following major criteria: (1) mitotic rate of > 50 mitoses per 50 high-power field (HPF), (2) any atypical mitoses, (3) any venous invasion. AONs are classified as borderline with uncertain malignant potential (BUMP) if they meet one to four of the following minor criteria: (1) size > 10 cm and/or weight > 200 g, (2) presence of necrosis, (3) sinusoidal invasion, (4) capsular invasion. AONs are categorized as benign in the absence of major or minor criteria. The reticulin algorithm characterizes malignancy based on the presence of an altered reticulin framework demonstrated by histological stain as basis and combined with at least one of the following features: (i) the presence of mitotic rate > 5 mitoses per 50 HPF, (ii) tumor necrosis, or (iii) vascular invasion. Lastly, the Helsinki scoring system is a point-based risk stratification system. It awards 3 points if there are greater than 5 mitoses per 50 HPF, 5 points for the presence of necrosis, and adds the numeric values form the Ki67 index. A Helsinki score of 0-8.5 suggests adrenal cortical adenoma and a score > 8.5 is suggestive of of adrenal cortical carcinoma. The tumor in our report met 3 of the LWB minor criteria (size > 10 cm, necrosis, capsular invasion) and was thus classified as an AON with BUMP. This tumor also exhibited a patchy loss of reticulin framework paired with the presence of tumor necrosis, which may be suggestive of malignancy—though unclear, per the reticulin algorithm. The only scoring system that was suggestive of malignant potential was the Helsinki system score of 16 (Ki67 of 11% and tumor necrosis), The prognosis of AONs is a function of malignant potential: 5-year survival was estimated to be 100% for benign tumors, 88% for borderline tumors, and 47% for malignant tumors, per LWB classification [11]. We managed this patient under the assumption that this tumor was AON with BUMP, following multidisciplinary discussions.

If an AON is classified as malignant or BUMP, adjuvant mitotane chemotherapy and adjuvant radiation therapy is considered based on extrapolated data from patients with ACC. The European Society of Endocrinology clinical guidelines recommends against use of adjuvant radiation therapy for stage I and II disease with negative surgical margins. Recommendation for adjuvant mitotane is limited to ACC patients with high risk of recurrence based on data from retrospective analyses that demonstrated improved recurrence-free and overall survival [12]. Indicators of high recurrence risk include presence of Ki67 staining in > 10% of cells, > 20 mitotic figure per 50 HPF, intraoperative tumor spillage, and large tumors with vascular or capsular invasion [9]. The tumor in our case demonstrated Ki67 > 10% and capsular invasion. However, shared decision-making resulted in surveillance without adjuvant mitotane or radiation, given negative surgical margins, favorable prognosis of AONs with BUMP, and the unclear benefit of such therapy on the oncocytic variant of adrenocortical carcinoma.

There are no standard post-operative surveillance recommendations for AONs. Given AON with BUMP, we opted for CT scan every 3 months for up to 5 years informed by clinical guidelines for ACC follow-up [7]. However, limited evidence from systematic reviews suggests that in comparison to ACCs, AONs with BUMP have markedly better prognosis and lower rate of recurrence [11]. As more cases of AONs are reported, the optimal surveillance schedule for AONs based on malignant potential may be determined.

We present a case of a 15 cm AON with borderline uncertain malignant potential treated with robotic surgery and review the post-operative decision-making management criteria. AONs are rare, typically benign tumors that can be safely treated with robotic-assisted adrenalectomy. Surgical pathology is part and parcel of diagnosis and determines then neoplasm’s malignant potential, which may inform both the initiation of adjuvant therapy and the post-operative surveillance regimen.

## Supplementary Information

**Additional file 1**. Video recorded robot-assisted adrenalectomy including the following steps: (1) dropping the colon and gaining access to retroperitoneum (2) retracting the adrenal mass up and creating the plane beneath the mass (3) dissecting the plane between pancreas (on the left) and the adrenal mass (on the right) (4) identifying renal and adrenal vein, clipping and cutting the adrenal vein (5) dissecting the plane between splenic artery and adrenal mass (6) dissecting the plane between adrenal mass and left kidney (7) cutting the final attachments (8) complete detachment of the mass.

## Data Availability

Not applicable.

## Abbreviations

ACC: Adrenal cortical carcinoma
AON: Adrenal oncocytic neoplasms
FDG-PET: 18 F-fluorodeoxyglucose positron emission tomography
LWB: Lin-Weiss-Bisceglia
BUMP: Borderline with uncertain malignant potential
